# Updates on Bone Grafts and Substitutes

**DOI:** 10.1055/s-0045-1814114

**Published:** 2025-12-30

**Authors:** Edgard Eduard Engel, Nelson Fabrício Gava, Mariana Avelino dos Santos, Leonardo Gomes Baldoino, Lucas Klarosk Ismael, Luis Felipe Miras Modolo

**Affiliations:** 1 Departament of Orthopedics and Traumatology, Hospital das Clínicas, Faculdade de Medicina de Ribeirão Preto, Universidade de São Paulo, Ribeirão Preto, SP, Brazil

**Keywords:** bone cements, bone regeneration, bone remodeling, bone substitutes, cimentos ósseos, regeneração óssea, remodelação óssea, substitutos ósseos

## Abstract

Bone defects caused by trauma, infections, neoplasms, and other conditions are commonly treated with autologous grafts, which are considered the gold standard due to their osteoinduction, osteoconduction, and osteogenesis properties. However, their use has limitations, including limited availability, donor-site morbidity, and increased surgical time. Alternatively, allogeneic grafts, xenografts, and synthetic bone substitutes, including ceramics, bioglasses, resins, and metals, have been developed and are often modified with osteoinductive elements, such as growth factors and bioinorganic ions. The ideal bone replacement should be biocompatible, bioabsorbable, mechanically resistant, porous, and capable of promoting osseointegration. Although synthetic substitutes have advanced, they have not yet achieved the effectiveness of autologous grafts, especially concerning osteointegration and economic viability. However, innovations in molecular biology, bone proteins, and gene therapies offer promising prospects for the development of new biomaterials. The current article introduces the reader to bone substitutes by presenting a classification of the materials used and the main characteristics of each group.

## Resumo

Os defeitos ósseos, causados por traumatismos, infecções, neoplasias e outras condições, são comumente tratados com enxerto autólogo, que é considerado o padrão-ouro devido às suas propriedades de osteoindução, osteocondução e osteogênese. No entanto, seu uso apresenta limitações, como a disponibilidade restrita, a morbidade no local doador e o aumento do tempo cirúrgico. Como alternativas, enxertos alógenos, xenógenos e substitutos ósseos sintéticos, que incluem cerâmicas, biovidros, resinas e metais, têm sido desenvolvidos, sendo frequentemente modificados com o acréscimo de elementos osteoindutores, como fatores de crescimento e íons bioinorgânicos. O substituto ósseo ideal deve ser biocompatível, bioabsorvível, mecanicamente resistente, poroso e capaz de promover osteointegração. Embora os substitutos sintéticos tenham avançado, ainda não alcançaram a eficácia do enxerto autólogo, principalmente no que se refere à osteointegração e à viabilidade econômica. Contudo, as inovações em biologia molecular, proteínas ósseas e terapias genéticas oferecem perspectivas promissoras para o desenvolvimento de novos biomateriais. Este artigo pretende apresentar ao leitor o tema dos substitutos ósseos, com uma classificação dos grupos de materiais utilizados e as principais características de cada grupo.

## Introduction

Bone defects are a clinical condition faced by orthopedists, neurosurgeons, head and neck surgeons, as well as dentists. In the orthopedic practice, these defects can result from high-energy trauma and major bone resections due to different pathologies, such as tumors, infections, loosening of prostheses, or complications in bone consolidation. The filling of bone defects aims to restore bone structure and, especially, mechanical strength. 1 2 The alternatives available for this filling can be grouped into grafts (biological materials) and substitutes (synthetic materials). 2

Autologous cancellous bone graft is recognized as the gold standard to treat bone defects or augment bone tissue. Each year worldwide, more than two million orthopedic surgeries are performed in which bone grafts are harvested, making it the second most common type of transplant, only behind blood transfusion. 2 Autologous bone is considered the ideal graft, as it is completely compatible and has all the indispensable characteristics for the osteointegration process, including osteoinduction, osteoconduction, and osteogenesis. 1 3 However, limited graft availability, potential donor-site morbidity, and the increased surgical time require the search for alternatives. 2 4

In bone defects, several types of biomaterials are being developed and tested, involving collaboration across areas such as medicine, biology, chemistry, engineering, and dentistry. 4 5

The goals of the current article are to present treatment alternatives for bone defects and their classification, and to discuss the theoretical principles of integrating bone substitutes and the future trends in the development of biomaterials.

## Definitions

Broadly speaking, grafts can be considered bone substitutes, as they meet the criteria for biomaterials. However, this term is most often used to describe synthetic materials derived from elements found in nature, and they can be classified into ceramics, polymers, bioglasses, and metals. 6 Therefore, *biomaterials* are natural or synthetic materials intended to replicate the form and/or function of different human or animal tissues, and they can act permanently or transiently. 7 Bone substitutes can be used to fill defects, replace bone segments, and to reinforce and stimulate bone consolidation at various body sites, percutaneously or openly. They should be structurally similar to bone, mechanically resistant, easy to handle, safe, and affordable. 8

Some features of bone substitutes are extremely important. They must present *biocompatibility* or at least biotolerance to ensure proper assimilation into biological tissues. *Biocompatibility* means that the organism accepts the material naturally, interacting with it at the metabolic level, while biotolerance indicates that it is accepted by the organism without causing significant adverse reactions, even if it does not elicit an ideal response. 3

*Osseointegration* is the main expected property of a bone substitute, characterized as the process of interdigitation between the host bone tissue and the implant. In this process, the recipient bone forms new bone tissue that penetrates the microscopic irregularities of the surrogate's surface, thereby stabilizing and fixing it in the insertion region. 1 8 This biological and mechanical integration is crucial to the implant's functionality and longevity. 2 6

Osteointegration depends on the following processes: osteogenesis, osteoconduction, and osteoinduction. *Osteogenesis* is the ability of the substitute to promote the formation of new bone tissue; 3 in practice, only the autologous graft has this property. *Osteoconduction* is the structural property of the material serving as physical support for the growth and regeneration of bone tissue. Osteoconductive material guides and facilitates the invasion and growth of bone cells, blood vessels, and bone matrix at sites where bone regeneration is occurring. 3 Such property is closely related to the material's surface characteristics and the dimensions of its pores, channels, or wells. *Osteoinduction* refers to a biomaterial's ability to stimulate undifferentiated cells to differentiate into osteoblasts, which are responsible for the formation of bone tissue 3 , thus promoting osteogenesis.

**Fig. 1:**
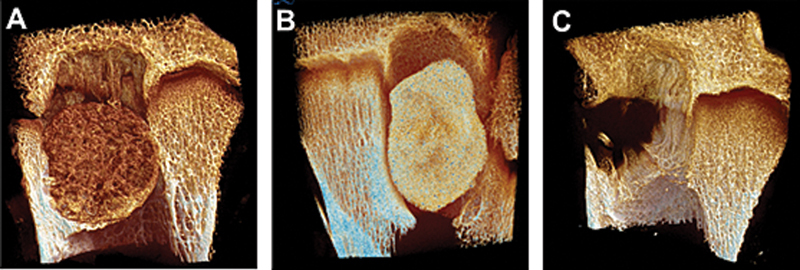
Comparison involving bone failures filled with porous bone substitute ( **A** ), solid cement block ( **B** ), and without filling ( **C** ), 3 months after surgery in rams, analyzed by microtomography. In ( **A** ), the contact between the bone and the surrogate is intimate, which indicates osseointegration. In ( **B** ), receptor bone resorption around the cement block and formation of fibrous tissue occurs. In the unfilled defect ( **C** ), the development of neoformed bone spicules seeking to invade the defect is observed.

Regarding the structure of the bone substitute, it is recommended that its morphology be analogous to that of the bone to be replaced, whether cortical or cancellous. However, integration depends on *pores* that enable invasion of newly-formed bone. For osteoblast invasion, pores between 80 μm and 200 μm are sufficient; for capillary invasion, pores between 300 μm and 500 μm are required, and this process will only be successful if the pores are interconnected. 9 10 11 Porosity also has a direct influence on the mechanical characteristics of the biomaterial; the more porous it is, the higher the modulus of elasticity, and the lower its mechanical strength 12 ( Figs. 1A–C ).

Two quantities are considered among the *mechanical characteristics* of the material: its strength, which is the load-bearing capacity of the material before fracture, and its elasticity, which is the ability of the material to deform temporarily under the load application and return to its original shape after removal of that load. Low resistance leads to fractures and mechanical failure of the implant. The discrepancy between the elasticities of the host bone and the implant, in turn, causes micromovements in the bone–biomaterial interface, preventing osseointegration. 11 12

The ability of the organism *to absorb* the substitute is highly desirable because it enables the gradual replacement by newly-formed bone tissue. 2 13 To maintain mechanical strength, the rate of resorption must be compatible with the speed of formation of new bone. In addition, the resorption rate is influenced by the porosity of the bone substitute, as this determines the area of contact between the material and the surrounding biological tissue. 2 14

The *presentation* of the substitute defines its best application. Moldable bone cements are best suited for filling misshapen spaces, while structured blocks are preferable in osteotomies. The *cost* and the amount of *material available* for the implant finalize the list of important properties of bone substitutes. The addition of growth hormones and the three-dimensional (3D) printing of metals such as tantalum increase the cost, while polymethylmethacrylate (PMMA) bone cement remains the most used in the clinical practice, mainly due to its low cost.

## Types of grafts

The *autologous graft* (also called *autogenous graft* or *autograft* ), taken from the patients themselves, is considered the ideal graft because it does not provoke an immunological reaction and exhibits osteoinduction, osteoconduction, and osteogenesis. 6 However, limited availability, potential morbidity of the donor site, additional surgery, increased operative time, increased blood loss, chronic pain in the donor region, dysesthesia, and infections impose limits on its use in the clinical practice. 1 2

Cancellous autologous grafts are often obtained from the iliac bone, tibia, olecranon, and calcaneus, which are poor in osteoblasts and osteocytes but are rich in primitive mesenchymal cells and osteoinducive factors, which facilitate revascularization and incorporation into the receptor site. 6 Cortical autologous grafts are obtained from the fibula and rib. 1 As the cortical graft contains a limited number of osteoprogenitor cells, integration occurs via osteoclast-mediated resorption, followed by apposition of newly-formed bone on the necrotic scaffold of the transplanted bone. This process is slower than the integration of cancellous bone. 6 The corticocancellous graft of the iliac crest combines the ease of integration of the cancellous bone with the structural integrity of the cortical bone. 15 Some cortical grafts (from the fibula and rib) and corticocancellous grafts (from the iliac crest) can be transplanted as flaps or vascularized grafts requiring microdissection or anastomosis. Medullary canal reaming, associated with irrigation and aspiration (reamer-irrigator-aspirator, RIA), is a method to obtain a greater volume of autologous graft with higher levels of growth factors and stem cells. 16

Biological alternatives to autologous grafts are *allogeneic grafts* ( *homologous grafts* or *allografts* ), which come from another individual of the same species, and *xenogeneic grafts* ( *xenografts* ), which come from another species.

Allogeneic grafts are available as bone segments and bone preparations. The segments are stored in tissue banks in various sizes and formats (total, cancellous, cortical), including for osteoarticular reconstructions. 17 Among the limitations of allografts are their lower incorporation capacity, greater risk of failure, potential immunogenicity and disease transmission, and the need for chemical preparation and storage methods such as lyophilization, freezing, or irradiation, which can affect their mechanical properties. 1 6 The most common processed allograft is the demineralized bone matrix (DBM), 6 which preserves growth factors but is limited by its lack of mechanical resistance, and it is more commonly used in dentistry. 18

Xenografts, which are derived from bovine, porcine, and equine bones, are processed to remove biological components and preserve hydroxyapatite (HA) and, occasionally, the collagen matrix. Their application in orthopedics has restrictions. 1 19 Extracted from other species, there is still chitosan, from shrimp shells, which is used to stimulate bone regeneration and cell differentiation, and HA extracted from corals. 2

## Types of bone substitutes

Bone substitutes can be technically grouped into four broad classes: ceramics, bioglasses, polymers, and metals. 15 *Ceramics* are widely used as bone substitutes due to their great biocompatibility. The great diversity in presentation creates some confusion regarding the nomenclature. The most commonly used material is calcium phosphate, which can be used in its crystalline form, which is found in animals (HA), or in the amorphous form, the most common example of which is beta-tricalcium phosphate (β-TCP). The combination of both is called *biphasic calcium phosphate* (BCP), and its presentation in cement form is known as *calcium phosphate cement* (CPC).

The main mineral component of bones and teeth, HA can be extracted from nature or synthetically produced in the form of particles or nanoparticles. 18 It has a low resorption rate, limited mechanical strength, and good osteoconductivity. That is why HA is widely used as an implant coating and less to fill bone defects. Notably, its nanocrystalline form demonstrates better biological performance, favoring cell adhesion and osteogenic differentiation. 20

Tricalcium phosphate, which is characterized by its rhombohedral structure and a lower calcium/phosphate ratio than HA, resorbs more rapidly (13–20 weeks). 13 Its interconnected micropores facilitate osteogenic cell invasion and angiogenesis, but its limited mechanical stability restricts its application under high-load conditions. 21 Despite this, β-TCP is widely researched and commercially available. 1

Biphasic calcium phosphate, a mixture of HA and β-TCP in varying proportions (40–60% of each), offers a balance between mechanical support and physiological bone resorption, making it a promising option for diverse clinical applications. 1 22

**Fig. 2:**
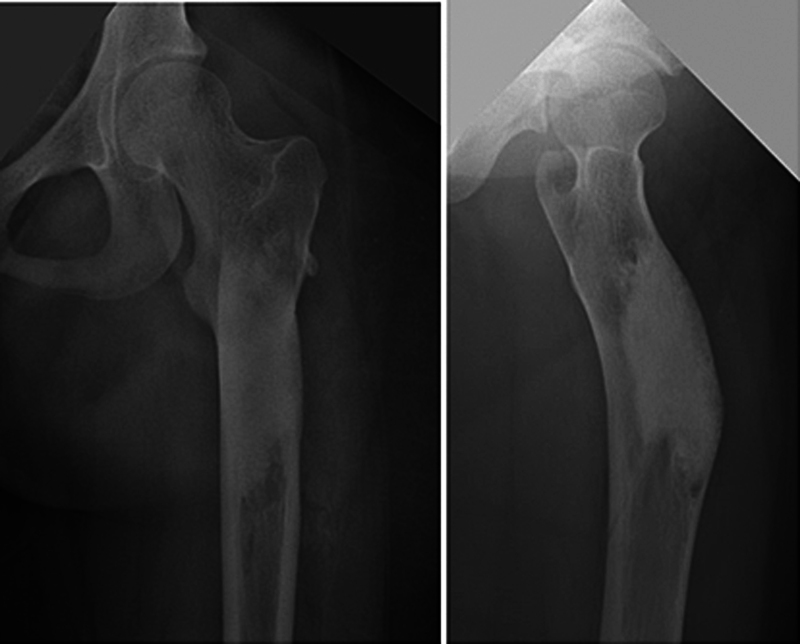
Femur radiograph showing the integration of calcium phosphate cement implanted 2 years before.

Calcium phosphate cements are moldable during the filling of bone defects. 23 Formed by mixing liquid and powdered components that precipitate in HA nanocrystals, they present low mechanical strength and porosity, limiting reabsorption and invasion by new bone. They are injectable and can carry biologically-active components such as osteoprogenitor cells and growth factors. 23 Due to their malleability, injectability, bioactivity, and biocompatibility, CPCs are a reality in the treatment of bone defects and highly promising for tissue engineering applications ( Fig. 2 ).

Calcium sulfate (plaster in its pure form) shows complete resorption in 6 to 8 weeks and weak stimulation of bone formation. To circumvent these restrictions, its application is often combined with DBM, which improves clinical outcomes. 1

*Bioglasses* are ceramics with a high concentration of silicate (45–52%), which are glasses and exhibit bioactivity due to their ability to strongly bind to the host bone. 24 Their bioactivity is believed to result from silicon ion leaching into body fluids; 24 these ions promote the formation of a layer of HA on the surface of the bioglass, attracting osteoprogenitor cells and initiating the gradual replacement of the material by the newly-formed bone. The high porosity and rapid absorption favor increased bone replacement but also raise local pH, which can inhibit bone remodeling and affect long-term effectiveness. 25 Because they are fragile and present low mechanical strength, their use becomes limited in areas that require load-bearing. 18

*Polymers* , many of which are also known as *resins* , are macromolecules formed by the polymerization of smaller molecules. The most commonly used natural polymers in bone tissue engineering are collagen, chitosan, gelatin, silk fibroin, alginate, cellulose, and starch. 26 Their high biocompatibility and low toxicity make them attractive for clinical applications, and their degradation products are generally non-toxic and easily reabsorbed by the body. A marketed Brazilian product is castor oil polyurethane, but its effectiveness is debated in the scientific literature. 27

Biodegradable synthetic polymers, such as polylactic acid (PLA), poly(lactic-co-glycolic acid) (PLGA), and polycaprolactone (PCL), stand out in tissue engineering due to their ability to adjust physical properties, including porosity, osteoconductivity, resorption rate, and final conformation. 1 5 6 These characteristics make them particularly suitable for customized implants, especially through techniques such as 3D printing. 5 In addition, the low immunogenicity of these materials is a significant advantage, contributing to their biological acceptance. However, during degradation, a reduction in local pH may occur, potentially compromising the adhesion of osteoprogenitor cells and the material's osteoconductive properties. 18 The use of biodegradable polymers is still relatively recent and requires further studies to consolidate the evidence and develop application guidelines.

A non-absorbable synthetic polymer, PMMA is widely used in orthopedics for the fixation of prostheses, 28 the filling of bone defects, mechanical reinforcement in cases of fragile bones, such as in vertebroplasties, to increase the mechanical resistance of osteosyntheses, 4 or to carry drugs such as antibiotics and chemotherapeutics. 29 30 When PMMA is introduced into bone defects, it forms a solid, compact mass that functions as a biotolerant spacer with no bone integration. 14 31

**Fig. 3:**
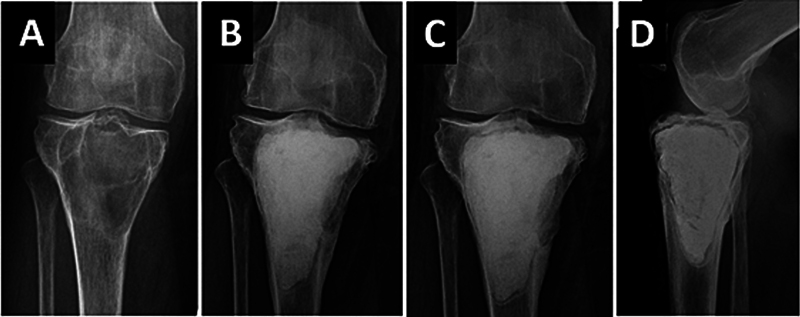
Radiographs of the proximal region of the tibia showing a giant cell tumor compromising the subchondral bone of the medial condyle ( **A** ). Two years ( **B** ) and 4 years ( **C** , **D** ) after surgery, bone resorption is observed around the cement block, more pronounced in the subchondral region. This reabsorption can lead to fracture, arthrosis, and even intra-articular exposure of the cement.

The PMMA cement offers advantages such as unlimited availability, immediate mechanical stability, ease of intraoperative molding, and affordability. However, its limitations include the exothermic reaction during formation, which can lead to overheating and bone necrosis. 32 Although its possible adjuvant effect in the treatment of aggressive tumors due to high temperature is discussed, this has not been proven yet. Bone cement loosening is a significant limitation, associated with bone resorption and fibrosis layer formation, easily detectable on radiographs, and usually stabilizes after 3 months. 33 A likely cause of this condition is the difference in elasticity between cement and cancellous bone, which leads to micromovements and bone resorption. 31 Away from the joints, the marginal sclerosis that surrounds the cement has no implications. However, in the subchondral region, bone resorption can result in fractures, joint incongruence, and arthrosis. 29 Finally, the definitive presence of cement prevents bone remodeling and hinders future interventions, which is its main disadvantage 14 ( Fig. 3 ).

*Metals* are well known to orthopedists and other professionals who perform osteosynthesis. Titanium (Ti) and chromium-cobalt (CoCr) steel plates, nails, and screws are biocompatible and present high mechanical strength and wear resistance. 4 The use of metals as bone substitutes became feasible with the advent of 3D printing (additive manufacturing, AM), enabling the production of defect-shaped parts from computed tomography scans. However, stress shielding, resulting from differences in metal and bone elasticity, is the main criticism to their use as bone substitutes, as it transfers mechanical stresses from the bone to the stiffer metal, reducing the mechanical osteoinducive stimulus on the bone and promoting its resorption. 4

To adjust the modulus of elasticity and promote osseointegration, these structures are produced with pores of variable size and quantity, and the metal alloy formulation can also be adjusted. Titanium is the most commonly used metal. The addition of tantalum (Ta) to Ti alloys further reduced the modulus of elasticity. 34 Biodegradable metals based on iron (Fe) and magnesium (Mg), which can already be produced by 3D printing, represent a promising innovation. Porous iron structures demonstrated resorption rates of 5 to 16% in 4 weeks, maintaining a modulus of elasticity comparable to that of cancellous bone in this period. Another approach under development is the creation of semi-absorbable Ti and Mg alloys, 35 evidencing a rapidly-evolving field of research with significant advances.

## Osteoinducers

Many osteoconductive bone substitutes can be used in combination with osteoinductors to enhance osteointegration. Simple impregnation of the bone substitute in bone-marrow aspirate may have an osteoinductive effect. 36 The first osteoinducers identified were bone morphogenetic proteins (BMPs), 1 a group of molecules classified as *growth factors* , belonging to the transforming growth factor β (TGF-β) superfamily. 6 They are produced by osteoblasts and play an important role in the recruitment and differentiation of osteoprogenitor cells in bone-formation sites. Despite several studies, the effectiveness of these components is controversial and lacks clinical evidence. The main limitations include the high solubility—which makes it difficult to fix the protein to the desired location—the high cost, and the risk of BMP escape, with the possibility of ectopic ossification in unwanted areas.

Among the growth factors, fibroblast growth factor (FGF) has already been used in clinical studies, although the mechanism of osteoinduction is not well understood. It is known that FGF is present throughout the fracture consolidation process, but its effect depends on the dose and the time of application. 6 Although studies have shown its positive effect, its clinical use has not yet been approved by regulatory agencies.

Vascular endothelial growth factor (VEGF) has two functions related to osteoinduction: direct effect on osteogenesis and on bone callus vascularization in fractures. 37 Concern regarding the induction of hemangioma formation and tumor recurrence, especially in those sensitive to VEGF, may restrict its use. 6

The parathyroid hormone (PTH) is commercially available and used to increase bone mass and reduce fractures in patients with osteoporosis. Studies 6 have shown that its use results in increased bone mass and shorter fracture consolidation time; however, there is no evidence that PTH initiates the bone remodeling or consolidation process.

Bioinorganic ions such as silicon, Mg, strontium, zinc, and copper play important roles in the body, including serving as enzyme cofactors, coenzymes, signaling molecules, and regulators of ion channels. Some of these functions are related to osteogenesis, and their osteoinducive properties have been widely studied and frequently confirmed. In addition, the incorporation of these ions in base materials presents advantages, such as low cost, longer shelf life, and probable reduction of risks compared to growth factors. However, robust evidence is still lacking to support regulatory approval of substitutes containing these ions as osteoinducers. 6 38

An osteoinducive factor that should not be overlooked is the mechanical factor. Cyclic bone deformation activates osteoblasts and promotes bone remodeling.

## Absence of filling

**Fig. 4:**
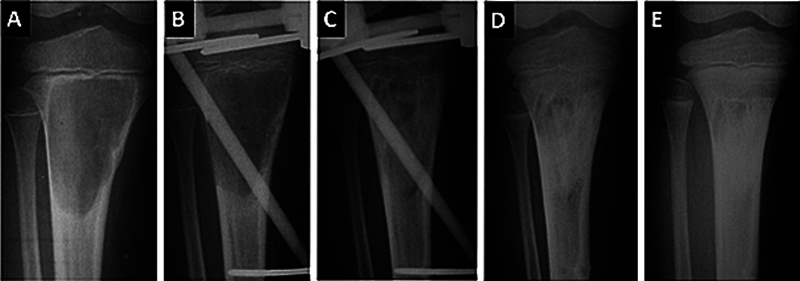
Serial radiographs of a 5-year-old patient with aneurysmal bone cyst in the tibia, adjacent to the growth cartilage ( **A** ). The patient underwent curettage and installation of a transphyseal external fixator, without filling the defect ( **B** ). Five months after surgery, at the time of fixator removal ( **C** ), progressive bone remodeling is observed, which continues 6 months ( **D** ) and 12 months ( **E** ) postoperatively. The growth cartilage remained intact.

Some authors 19 30 consider bone defect filling unnecessary in certain clinical situations because they observe high rates of bone neoformation induced by mechanical stimulation. In these cases, remodeled bone appears different from normal bone but may still have sufficient mechanical strength to support the functional load. The risk of fracture before complete bone remodeling is mitigated by prophylactic osteosynthesis, which can prevent mechanical complications and enable early load support, a situation not always possible with bone substitutes. 19 Therefore, bone grafts and substitutes can only be considered effective if their remodeling potential is greater than that of natural bone remodeling ( Fig. 4 ).

## Advances

The emergence of various synthetic bone substitutes offers a wide variety of options. However, the treatment outcome is still incomparable to that of autologous bone graft in terms of bone healing quality and time. Overall, the effectiveness of the substitutes available still depends on a favorable site and the size of the defects.

The most significant recent advances in osteoinducers involve increased production and reduced cost of growth factors through recombinant technology. Recombinant human BMPs (rhBMPs), which have demonstrated reasonable osteoinductive activity, 39 as well as the understanding of the osteoinducive effect of bioinorganic ions is an important and promising line of research. 6

The development of metal substitutes with mechanical properties compatible with those of cortical bone, low toxicity, and large and interconnected pores, enabling osteointegration, has shown good results. The absorbable metals that can be produced by 3D printing 4 arouse great interest, as does 3D bioprinting with active cells such as osteoblasts and osteoclasts. 35

The application of tissue engineering to musculoskeletal pathology has uncovered potential treatment strategies. Regional gene therapy involves the local implantation of nucleic acids or cells genetically modified to direct specific protein expression, and it has shown promise as a bone regeneration technique. 40

With recent advances in tissue engineering, a new approach has emerged focused on “tissue regeneration by natural tissues,” rather than “tissue replacement by biomaterials.” Coculture and triculture of different cell types in CPC structures offer great potential to promote vascularization in bone regeneration, especially in the treatment of large bone defects. 6 However, more studies are required to validate these approaches and elucidate the underlying mechanisms to advance tissue engineering and regenerative medicine.

## Conclusion

The treatment of bone defects is a major challenge for surgeons. The gold standard is still the autologous graft, but its use faces restrictions such as limited availability and surgical complications.

Despite technological advances in this field, substitutes have not yet achieved the clinical effectiveness of the autologous graft. Whether due to bioavailability limitations, osteoinduction, osteoconduction, or cost, the search for a bone substitute that reconciles all these variables remains a challenge for the scientific community.

The high number of bone replacements worldwide, coupled with the commercial potential of an effective biomaterial, has driven significant advances in the field. New technologies in molecular biology, AM, gene therapies, and tissue bioengineering offer a promising horizon for the development of bone substitutes.
